# Automated algorithm for calculation of setup corrections and planning target volume margins for offline image‐guided radiotherapy protocols

**DOI:** 10.1002/acm2.13291

**Published:** 2021-06-09

**Authors:** Auwal Abubakar, Nada Alia M. Zamri, Shazril Imran Shaukat, Hafiz Mohd Zin

**Affiliations:** ^1^ Advanced Medical and Dental Institute (AMDI) Universiti Sains Malaysia Kepala Batas 13200 Malaysia; ^2^ Department of Medical Radiography, Faculty of Allied Health Sciences, College of Medical Sciences University of Maiduguri Maiduguri Nigeria

**Keywords:** automation, CBCT, IGRT offline correction protocol, PTV margin, setup error

## Abstract

**Purpose:**

Each radiotherapy center should have a site‐specific planning target volume (PTV) margins and image‐guided (IG) radiotherapy (IGRT) correction protocols to compensate for the geometric errors that can occur during treatment. This study developed an automated algorithm for the calculation and evaluation of these parameters from cone beam computed tomography (CBCT)‐based IG‐intensity modulated radiotherapy (IG‐IMRT) treatment.

**Methods and materials:**

A MATLAB algorithm was developed to extract the setup errors in three translational directions (*x*, *y*, and *z*) from the data logged by the CBCT system during treatment delivery. The algorithm also calculates the resulted population setup error and PTV margin based on the van Herk margin recipe and subsequently estimates their respective values for no action level (NAL) and extended no action level (eNAL) offline correction protocols. The algorithm was tested on 25 head and neck cancer (HNC) patients treated using IG‐IMRT.

**Results:**

The algorithms calculated that the HNC patients require a PTV margin of 3.1, 2.7, and 3.2 mm in the *x*‐, *y*‐, and *z*‐direction, respectively, without IGRT. The margin can be reduced to 2.0, 2.2, and 3.0 mm in the *x*‐, *y*‐, and *z*‐direction, respectively, with NAL and 1.6, 1.7, and 2.2 mm in the *x*‐, *y*‐, and *z*‐direction, respectively, with eNAL protocol. The results obtained were verified to be the same with the margins calculated using an Excel spreadsheet. The algorithm calculates the weekly offline setup error correction values automatically and reduces the risk of input data error observed in the spreadsheet.

**Conclusions:**

In conclusion, the algorithm provides an automated method for optimization and reduction of PTV margin using logged setup errors from CBCT‐based IGRT.

**Highlights**

- Automated algorithm provides a fast and error free offline setup corrections during image‐guided radiotherapy (IGRT).
- A reduction of a margin of up to 48% for head and neck cancer cases if (eNAL) protocol is used.
- Institutional based margin can be calculated using the automated algorithm.

## 1 INTRODUCTION

Geometrical uncertainties during patient setup is a limiting factor in achieving the high precision and accuracy required in radiation delivery during radiotherapy treatment.^1^ Even though immobilization is in place, uncertainties may still arise due to motion from breathing, swallowing, or coughing in some instances.^2^, ^3^ Additional uncertainties could also occur as patients tend to move after positioning due to pains or anxiety.^4^ It is also challenging to reproduce patient setup daily in patients, particularly with deformed physical characteristics such as kyphosis or scoliosis.^4^ Other uncertainties could be due to tumor regression in response to the treatment and changes in soft tissues due to weight loss as commonly seen towards the last few weeks of the treatment.^5^, ^6^, ^7^ In order to ensure precise and reproducible patient setup for the accuracy of treatment delivery, image‐guided radiotherapy (IGRT) is adopted.

IGRT involves imaging the target location and patient setup and corrects for any changes in patient and target position before each treatment session.^4^, ^8^, ^9^ The use of cone‐beam CT (CBCT) for IGRT has become widely available in the last decade.^9^, ^10^, ^11^ It provides fast and accurate displacement information of each patient in the translational directions, and also rotational directions for some centers with the rotational couch.^12^, ^13^ IGRT is also used to calculate systematic and random setup errors and, subsequently, the planning target volume (PTV) margin for patients treated for the same anatomical site.^14^, ^15^, ^16^ This may reduce the PTV margin, allowing possible dose escalation to tumor volume and enabling surrounding healthy tissue to be spared.^17^, ^18^, ^19^ The previous study conducted by Guckenberger *et al*. in which head and neck cancer (HNC) patients were immobilized by individualized thermoplastic mask or Scotch cast mask revealed that translational setup error could be as high as −5.4 mm.^20^ Further reduction of the PTV margin to 3 mm could be achieved when daily IGRT is employed for HNC patients treated with intensity modulated radiotherapy (IMRT).^21^, ^22^ Thus, the utilization of IGRT during IMRT may reduce the risk of geometric miss among HNC patients.

Online IGRT protocol involves the daily acquisition of verification images and correction prior to the treatment delivery. The verification images are acquired with the patient in the treatment position and matched with the computed tomography (CT) simulation reference images. The protocol measures the difference as setup error, and the correction is made by shifting the patient's position using the automatic setup function in the linac control system software before the treatment is delivered.^23^, ^24^ The online protocol is effective in the reduction of both systematic and random errors.

The offline protocol involves measurement in a number of imaged fractions, and then, the correction is applied to the subsequent fractions. This protocol reduces only systematic but not random error. The protocol also has the potential to reduce imaging dose to the patient due to less frequent imaging. The common offline protocols include no action level (NAL) and extended no action level (eNAL) protocols.^25^, ^26^ NAL protocol performed the correction after three to four fractions. The mean error for the first few fractions is applied to all the subsequent fractions as a correction.^27^, ^28^ eNAL protocol involves imaging in the first three to four fractions and additional weekly imaging.^27^, ^29^ This protocol effectively detects systematic setup changes and trends that might occur in subsequent fractions, which is not detectable using NAL protocol.^25^ However, the frequency of imaging sessions is higher for eNAL protocol and thus, resulting in greater workload and imaging dose than NAL protocol.

The offline protocols are effective in terms of determining systematic setup error and margin reduction. Additionally, the protocols require less frequent imaging compared with the online protocol, and that could potentially reduce the patient imaging dose and radiation therapist workload. Despite that, offline protocols are not widely implemented.^30^ This is mainly because offline protocol involves manual extraction and recording of the individual fraction setup error and subsequent calculations of the corrections values as this is not available in the IGRT system of the linear accelerator. The manual process is tedious, time consuming, and could be prone to data input errors. The IGRT National Implementation Group, UK, recommends that every radiotherapy center should develop their PTV margin as every center could vary due to the use of different immobilization devices, correction strategies, and experience of the radiation therapist, which eventually impact on the setup error and ultimately the PTV margin.^31^, ^32^, ^33^, ^34^, ^35^

In this paper, we focused on the development of an algorithm that provides an automated method for the calculation of the correction values for the offline protocols (NAL and eNAL). This is hoped to overcome the offline protocols' limitation and facilitate the development of institutional based PTV margins. The algorithm also calculates the PTV margin using the van Herk's formula, and the percentage reduction is achieved with NAL and eNAL protocols among HNC patients treated with IG‐IMRT. The margin recipe is widely adopted to calculate PTV margins, and it is determined by combining the standard deviations of the systematic and random errors.

## 2 MATERIALS AND METHODS

### 2.A Patients characteristics and CT simulation

Twenty‐five HNC IMRT patients were included in this study. These include nine nasopharyngeal, seven larynx, four oropharynx, three oral cavity, and two hypopharynx carcinoma. The patients were 9 females and 16 males with an age range between 23 and 71 years.

Patients were immobilized in a supine position using HeadSTEP iFRAME immobilization device and iCAST thermoplastic mask that covers the head, neck, and shoulders. Target volumes were delineated and contoured according to International Commission on Radiation Units and Measurements (ICRU) reports 62.^14^ For HNCs in our institutions, only CT images are used for contouring and delineation. Positron emission tomography (PET)/CT and magnetic resonance imaging (MRI) are used for reference but is not fused into the treatment planning system; hence, contouring is solely based on CT‐based delineation.

Dose calculation and optimization of the IMRT delivery were performed using the Monaco treatment planning system (Elekta Medical Systems, Crawley, UK). Beam plan for all the cases studied was with seven gantry angles as follows: 0°, 51°, 102°, 153, 204°, 255°, and 306°. The total dose delivered was in the range of 60–70 Gy per patient with 2‐Gy dose delivered for every fraction. Patients were treated with one fraction daily for 5 days a week, with a total of 30–35 fractions per patient.

### 2.B Treatment delivery

**Fig. 1:**
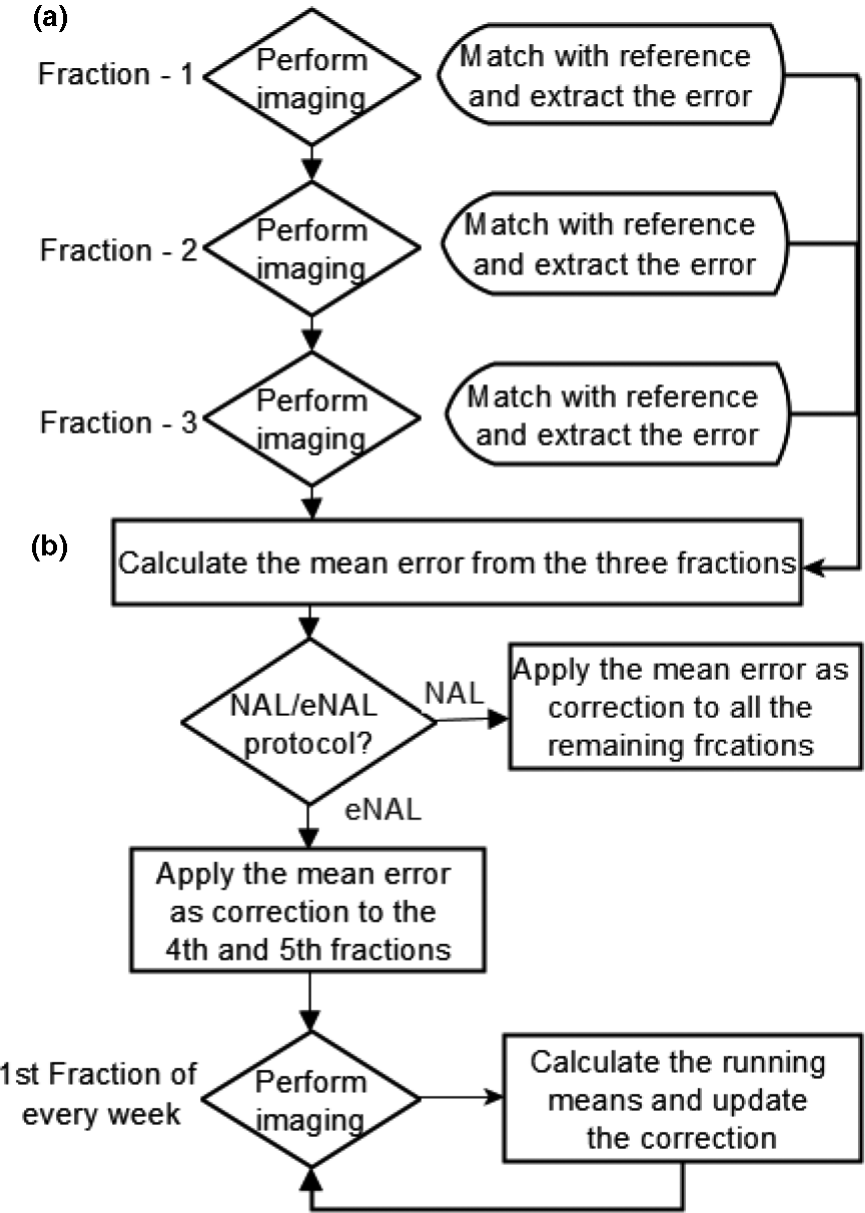
Flow chart for the cone beam computed tomography (CBCT) image registration, no action level (NAL), and extended no action level (eNAL) offline image‐guided radiotherapy (IGRT) protocols.

The patients were treated using Elekta Synergy linear accelerator (Elekta Medical Systems, Crawley, UK), equipped with the X‐ray volume imaging (XVI) CBCT system to acquire and process the CBCT images. The pretreatment CBCT images were acquired after the patient setup in the first three fractions and subsequently, once per week. The CBCT images were acquired after aligning the in‐room lasers with the corresponding marks drawn on the thermoplastic mask. The images were acquired using 100 kV, 10 mA, and 10 ms. A total of 183 frames were acquired for a total gantry rotation of 200°. The collimator used was S20 to provide a field of view of 26 cm in diameter and 26 cm in length. All CBCT images were registered to the planning CT images using automatic bone matching (correlation coefficient algorithm) in the XVI software on the CBCT control computer to obtain the setup error in the three translational directions (Fig. 1a). Cervical spine is used for bone matching. A moderate size of region of interest (ROI) is selected to include the C‐spine. The bone matching is checked jointly by two radiation therapists. In the event of significant deviation in the error, the radiation therapists would notify the treating oncologist. In this report, *x*‐, *y*‐, and *z*‐axes are used to describe right‐left, superior‐inferior, and anterior–posterior direction, respectively, as defined in the IEC 61217 standard.^36^

The setup errors were defined as the offset between CBCT and planning CT in three translational directions. For each patient, the setup error for every fraction in which imaging was performed in all the three translational directions is automatically saved in a text file and stored on the CBCT system.

### 2.C The tools for analysis

#### 2.C.1 The automated MATLAB algorithm

**Fig. 2:**
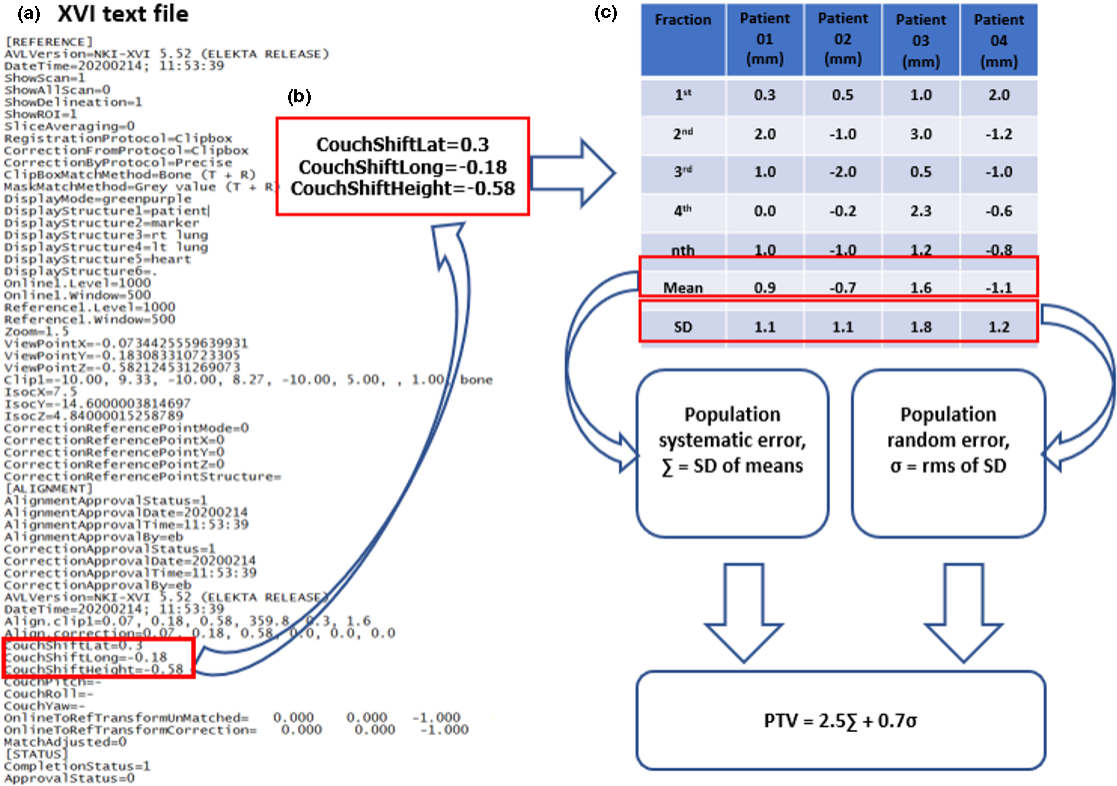
Illustration of planning target volume (PTV) calculation using the automated algorithm.

The workflow of the algorithm is illustrated in Fig. 2. The algorithm was developed using MATLAB (MathWorks Inc, Natick MA) to extract setup error data from the XVI text file. The script is provided in Supporting information Appendix S1. The entire MATLAB script and sample XVI files are available online (https://github.com/hafizmz/IGRTmargins). Figure 2a shows a sample content of the XVI text. The file contains large amount of information, including the registration method, displayed structures, image zoom, and the shift data. The logged XVI text files are located in the XVI computer and could be traced and identified in the “Reconstruction” subfolder as .INI file. The algorithm reads all the .INI text files in the subfolders belonging to a patient and searched the following keywords: “CouchShiftLat,” “CouchShiftLong,” and “CouchShiftHeigh” that represents the translational setup error in *x*‐, *y*‐, and *z*‐axes, respectively. Subsequently, it extracts the translational setup errors' values and calculates the individual systematic and random errors for each patient as shown in (Fig. 2b). The table in Fig. 2c shows an example of the calculation for four patients for one translational setup error direction. The setup errors, individual random error, and systematic error are automatically saved as a MATLAB variable. The data extraction and calculation are repeated for each patient by moving the process to the subsequent folder containing the XVI files for each patient. Afterwards, the algorithm loads the setup errors from the data variable saved in MATLAB and calculates the population error and PTV margin for no correction protocol, NAL, and eNAL protocols. In addition, the percentage reduction in PTV margin achieved with NAL and eNAL protocols can be compared with no correction protocols.

To simulate the NAL and eNAL protocols, at first, the algorithm calculates the mean setup error values for the first three fractions (Fig. 1b). This represents the correction value and is applied as a correction to all the subsequent fractions for NAL protocol since imaging is only performed during the first three fractions. For eNAL protocol, which involves additional weekly imaging after the first three fractions, the correction is applied to the fourth and fifth fractions. Subsequently, the algorithm re‐calculates the running mean of the error values at the first fraction of every week and applied as correction to the same week's remaining fractions.

The algorithm calculates the individual systematic and random error as the mean and standard deviation of a patient's setup errors throughout the treatment course. Population systematic error, ∑, was calculated as the standard deviation of the individual systematic error, while the population random error, σ was calculated as the root mean square of the individual random error as shown in Fig. 2. These parameters were used to calculate the PTV margin using van Herk equation (PTV=2.5∑+0.7σ).^37^

#### 2.C.2 The Excel spreadsheet

An Excel spreadsheet was also used to manually enter the setup error values in the *x*‐, *y*‐, and *z*‐axes for each patient in a separate worksheet. The individual and systematic random errors were calculated for each patient. Another worksheet was programmed to calculate the PTV margins for NAL and eNAL protocols. The Excel spreadsheet is provided in Supporting information Appendix S2.

## 3 RESULTS

### 3.A The automated MATLAB algorithm vs the MS Excel spreadsheet

The in‐house developed MATLAB (MathWorks Inc., Natick, MA, USA) algorithm reads out the XVI text files from a total of 231 pretreatment CBCT imaging done on 25 HNC IMRT patients. The algorithm completes the data extraction process and computes the *x*‐, *y*‐, and *z*‐axes setup errors within a few milliseconds for each patient, then another few more milliseconds for calculation of the population errors and PTV margin. The computer is a 64‐bit Windows 10 Pro, with an Intel® Core™ i7‐6700HQ 2.60 GHz CPU, and 32 GB of RAM, running MATLAB® (R2020a).

Excel spreadsheet requires the setup error data to be extracted manually and keyed in on the spreadsheet. Comparison of the results from the automated algorithm with the Excel spreadsheet revealed some discrepancies in the results. Further scrutiny of the results and raw data found that the Excel spreadsheet's errors were due to the calculation or formula error and data entry error. This could be due to human error inherent to the data input process. However, the error could be user dependent and thus, might be error free for a meticulous user. The limitation associated with the approached used in the spreadsheet method could be overcome by MATLAB due to its programming flexibility. For example, the number of imaging varies for breast (15–16 fractions) compared with the head and neck IMRT (30–35 fractions). The algorithm automatically detects the number of text files available depending on the number of CBCT imaging performed. This allows complete automation of the whole process and eliminates the chances of error in the data extraction, data entry, and calculations associated with the traditional spreadsheet method. Also, it significantly reduces the analysis time. This is more efficient than the spreadsheet approach in terms of the speed and accuracy of the calculations. The automated algorithm's efficiency may encourage individual center to develop an institutional PTV margin and offline CBCT imaging protocol that suit a busy radiotherapy center.

### 3.B Individual systematic and random setup errors

**Fig. 3:**
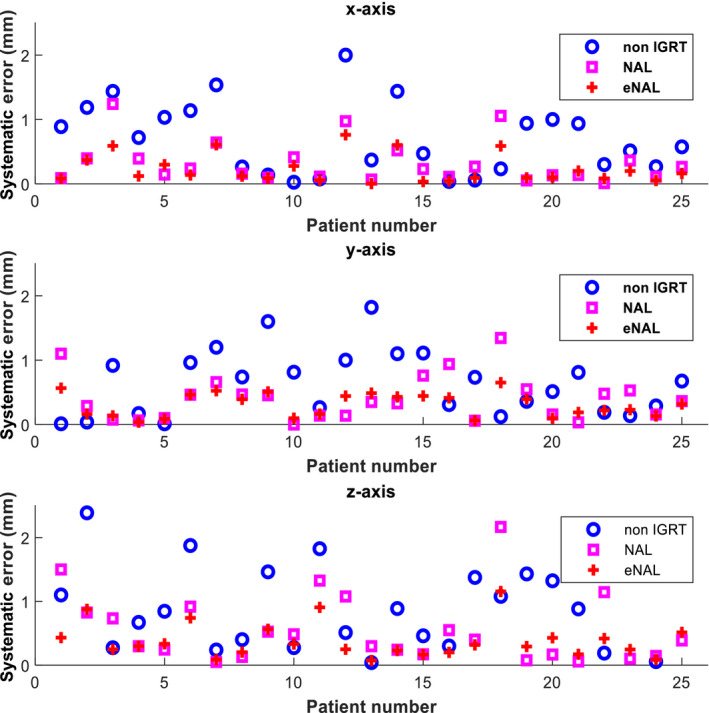
Individual systematic error along three translational dimensions. eNAL, extended no action level; IGRT, image‐guided radiotherapy; NAL, no action level.

**Fig. 4:**
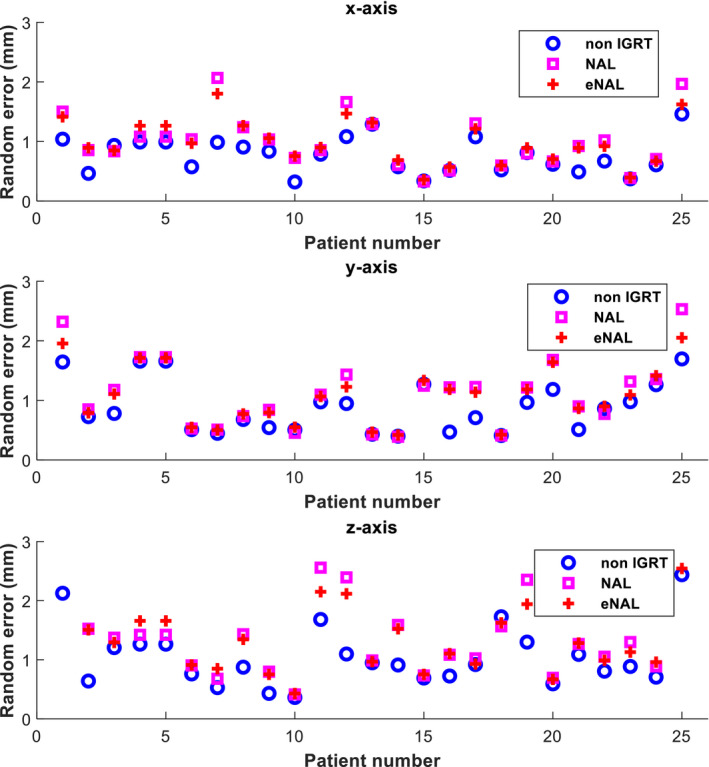
Individual random error along three translational dimensions.

Figures 3 and 4 show the systematic and random errors calculated for each patient using the algorithm. The plots show that both NAL and eNAL offline IGRT protocols could reduce systematic individual setup errors to different degrees relative to no correction protocol. The setup errors were larger for no correction protocol in which no positioning corrections were applied. For all the three protocols, the largest error occurred in the *z*‐axis. The largest error seen for no correction protocol, NAL, and eNAL protocols were 2.4, 2.2, and 1.2 mm, respectively. This showed that the largest reduction achieved was using the eNAL protocol. This could be associated with an increased number of imaging attributed to the eNAL protocol. Unlike the NAL protocol, the eNAL protocol involves additional weekly follow‐up imaging, which allows for the detection of time trend transition or sudden changes that might occur during the treatment course.^25^ The changes might likely occur among HNC patients treated with IMRT as the treatment course lasts for up to 7 weeks. Similarly, the largest random setup error was observed in *z*‐axis for all the three protocols. The largest errors seen were 1.1, 1.6, and 1.4 mm for no correction protocol, NAL, and eNAL protocols, respectively. This affirms the findings reported in the literature that offline protocols effectively reduce systematic error but not random error.^27^

### 3.C Population systematic and random setup error

**Fig. 5:**
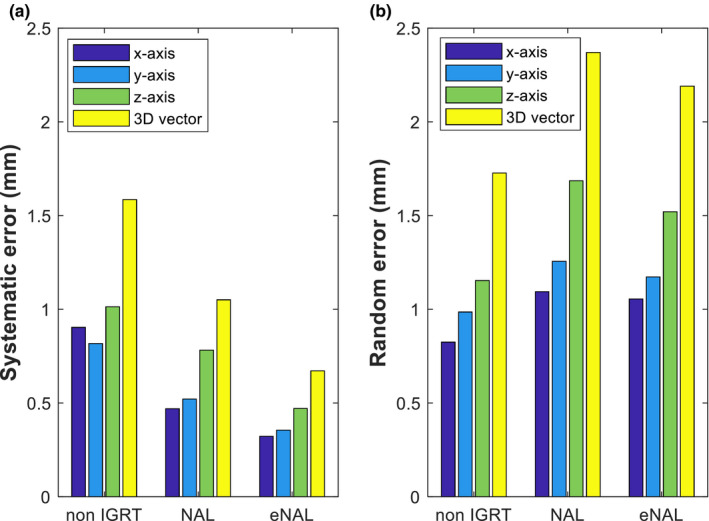
Results of the population systematic (a) and random (b) setup errors.

The population systematic and random setup errors were calculated from the corresponding individual setup errors. The results for the population systematic and random setup error are presented in Fig. 5. The population setup error revealed in this study shows that reductions in systematic errors were achieved using NAL and eNAL offline protocols compared to the no correction protocol. The largest population systematic error values were 1.0, 0.8, and 0.5 mm for no correction protocol, NAL, and eNAL protocol, respectively. The corresponding 3D vectors were 1.6, 1.1, and 0.7 mm.

### 3.D PTV margin and percentage reduction

**Fig. 6:**
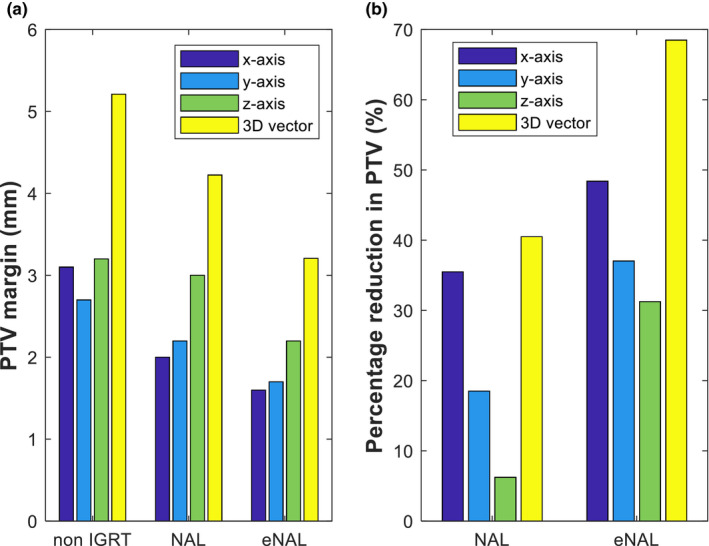
Planning target volume (PTV) margin (a) and the percentage reduction achieved (b) for various protocols.

Figure 6a shows that eNAL provides a smaller PTV margin compared with NAL. A greater percentage reduction in PTV for eNAL of up to 48% which is along the *z*‐axis and 68.5% for 3D vector is shown in Fig. 6b.

## 4 DISCUSSION

### 4.A The automated algorithm

This work develops an automated method for the calculation of correction values for NAL and eNAL offline IGRT protocol and their respective PTV margins. Offline IGRT correction protocols are introduced to reduce systematic setup error at less workload and imaging dose due to reduced imaging sessions. However, the correction value for offline protocols is not provided in the CBCT software on the linac system. The radiation therapists are left with no option than to embrace the manual calculation method to establish the patient's correction value if the offline protocol must be implemented. This limitation might be the major obstacle towards the wide implementation of the offline protocols in many radiotherapy departments. It is recommended that every radiotherapy center evaluates the setup error from at least 20 patients for each common anatomical site to establish their local PTV margin. This should be repeated after every 2 years or with a change in the protocols, immobilization devices, or linac machine.

The use of an Excel spreadsheet is the common method for the PTV margin calculation. However, this could be prone to some data entry and calculation errors. Also, it is time consuming and could be difficult to implement in the ever‐busy radiotherapy centers.

The algorithm developed in this study allowed for the calculation of individual and population setup error and PTV margin for offline (NAL and eNAL) and non‐IGRT protocols. Hopefully, this will overcome the hindrance towards the wide implementation of the offline protocol and facilitates the development of institutional‐based PTV margins. The algorithm was tested and validated using the traditional spreadsheet method on data from HNC patients treated using IMRT in our center.

### 4.B Population systematic and random setup error

The population error results show that the reduction in error values was achieved with the IGRT protocols compared with the no correction protocol. The result was not unexpected as various studies indicated that the use of IGRT reduces setup error in HNC patients.^4^, ^38^, ^39^ Also, the eNAL protocol was shown to be more effective in the reduction of population setup error compared with the NAL protocol. The reduction seen in eNAL compared with NAL could be due to the ability of the eNAL to detect and corrects setup errors that might occur in later fractions due to time trend transition or sudden changes during the treatment.^25^ These include errors due to weight loss, which could increase patient movement chances within the mask or due to tumor shrinkage in response to the treatment. The NAL protocol lacks the capacity to detect these errors as it employs imaging only during the first few fractions of the treatment. The larger error consistently seen on the *z*‐axis could be due to couch sag. In contrast, no reduction in population random error was seen with any of the offline protocols. This is not surprising as the offline protocols are meant to reduce systematic setup error.^25^, ^27^ Reduction in systematic error would significantly impact the PTV margin as it remains the major contributor in the margin formula. The systematic error shifts the cumulative dose distribution away from the target volume and thus appears to be more important than the random error, which only blurs the dose distribution.^16^

### 4.C PTV margin and percentage reduction

The PTV margin results follow a similar pattern as the population systematic error with the largest margin recorded in *z*‐direction for all the three protocols investigated (Fig. 6). This corresponds to the results from a similar study, which reported that the largest setup error and PTV margins were seen along the *z*‐axis.^40^ This study shows that the HNC patients require a PTV margin of 3.1, 2.7, and 3.2 mm, in the *x*‐, *y*‐, and *z*‐direction, respectively, without IGRT. The margin was found to reduce to 2.0, 2.2, and 3.0 mm, respectively, with NAL and 1.6, 1.7, and 2.2 mm, respectively, with eNAL protocol. This implies that a reduction in the PTV margin was achieved with both NAL and eNAL offline protocols. Although there is a dearth of literature on the use of eNAL protocol in HNC patients, there are a few PTV values reported for NAL protocol among the HNC patients. Lozano et al. reported PTV margins of 3.3, 0.6, and 2.3 mm, in *x*‐, *y*‐, and *z*‐axis among HNC patients were treated using NAL protocol.^41^ A similar result was reported in which the *x*‐, *y*‐, and the *z*‐axis PTV values were 3.8, 3.4, and 5.1 mm, respectively.^40^ The slight variations in the PTV margin seen could be due to the variation of the image guidance technique used in the studies. While our study involved the use of CBCT, the previous studies used EPID^41^ and helical tomotherapy.^40^ The eNAL was shown to allow greater reduction than the NAL protocol. This agrees with a simulation study and clinical data that revealed that the eNAL protocol supersedes NAL protocol to reduce PTV margin.^25^, ^42^

The application of the automated calculation algorithm for the setup error and PTV margin developed in this study is limited to data obtained from the CBCT IGRT system. It cannot be used with other IGRT systems such as EPID. Also, it does not consider setup error in the rotational direction as well as the dosimetric consequences that would occur due to the variation in the setup error and the PTV margins for different protocols. Although this work focused on the setup error there are other sources of uncertainties that contribute to systematic error and, therefore, could be considered for an extensive PTV margin estimation. Target volume delineation error is one of the largest uncertainties in the radiotherapy treatment process.^43^, ^44^ The use of multi‐modality imaging approach and implementation of consensus guideline and training may help to reduce this error.^45^, ^46^ Other causes of these uncertainties include isocenter misalignment between linac and imaging modality, image resolution, collimator and gantry angle error, field edge and MLC edge alignment error, and couch precision. These parameters are routinely checked to be within the designated tolerance. The uncertainty contribution for each parameter could also be considered in the PTV margin calculation.

## 5 CONCLUSION

In this paper, we reported the first “automated” algorithm for the calculation of the correction values for offline (NAL and eNAL) IGRT protocols. We have developed a practical and “automated” algorithm for the calculation of setup error correction for NAL and eNAL offline IGRT protocols. The algorithm also allows for automated calculation of the population setup errors and PTV margin for the no correction protocol, NAL, and eNAL offline protocols from the same dataset. The algorithm would allow the choice of the most effective protocols in terms of setup error and PTV margin reduction for a specific anatomical site for a radiotherapy center. The algorithm was tested on HNC patients, and the results show that both NAL and eNAL offline protocols were effective in terms of systematic error and PTV margin reduction, with eNAL being the most effective.

## CONFLICT OF INTEREST

The authors declare that they have no known competing financial interests or personal relationships that could have appeared to influence the work reported in this paper.

## DATA SHARING STATEMENT

All data generated and analyzed during this study are included in this published article (and its supplementary information files).

Authors responsible for statistical analysis: Hafiz Mohd Zin; hafiz.zin@physics.org.

## Supporting information

Appendix S1
Appendix S2

## Data Availability

The data that support the findings of this study are available on request from the corresponding author. The data are not publicly available due to privacy or ethical restrictions.
